# *Thymus algeriensis* and *Thymus fontanesii* exert neuroprotective effect against chronic constriction injury-induced neuropathic pain in rats

**DOI:** 10.1038/s41598-020-77424-0

**Published:** 2020-11-25

**Authors:** Samar Rezq, Amira E. Alsemeh, Luigi D’Elia, Assem M. El-Shazly, Daria Maria Monti, Mansour Sobeh, Mona F. Mahmoud

**Affiliations:** 1 Department of Pharmacology and Toxicology, Faculty of Pharmacy, Zagazig University, Zagazig, 44519 Egypt; 2 Department of Anatomy and Embryology, Faculty of Medicine, Zagazig University, Zagazig, Egypt; 3 Department of Chemical Sciences, University of Naples Federico II, Complesso Universitario Monte Sant’Angelo, via Cinthia 4, 80126 Naples, Italy; 4 Department of Pharmacognosy, Faculty of Pharmacy, Zagazig University, Zagazig, 44519 Egypt; 5 AgroBioSciences Research, Mohammed VI Polytechnic University, Lot 660–Hay MoulayRachid, 43150 Ben-Guerir, Morocco

**Keywords:** Biochemistry, Drug discovery, Neuroscience, Neurology

## Abstract

We have previously demonstrated that the *Thymus algeriensis* and *Thymus fontanesii* extracts have powerful anti-inflammatory, antipyretic, and analgesic effects against acute pain models. We profiled their chemical composition and found many phenolic acids, flavonoids, and phenolic diterpenes. In this work, we investigated their antioxidant properties on HaCaT cells exposed to UVA-induced oxidative stress and examined their effects against chronic neuropathic pain and the underlying mechanisms. Through a rat chronic constriction injury (CCI) model, we induced chronic neuropathic pain by placing 4 loose ligatures around the right sciatic nerve for 14 days. Thermal and mechanical hyperalgesia in addition to cold and dynamic allodynia were tested on the day before surgery and on the 7th and 14th post-surgery days. Key markers of the nitrosative and oxidative stresses, in addition to markers of inflammation, were measured at day 14 post surgery. Histopathological examination and immunostaining of both synaptophysin and caspase-3 of sciatic nerve and brain stem were also performed. Results of this study showed that *T. algeriensis* extract suppresses UVA oxidative stress in HaCaT cells via activation of the Nrf-2 pathway. Both extracts attenuated hyperalgesia and allodynia at 7- and 14-days post-surgery with more prominent effects at day 14 of surgery. Their protective effects against neuropathic pain were mediated by inhibiting NOX-1, iNOS, by increasing the enzyme activity of catalase, and inhibition of inflammatory mediators, NF-κB, TNF-α, lipoxygenase, COX-2 enzymes, and PGE2. Furthermore, they improved deleterious structural changes of the brainstem and sciatic nerve. They also attenuated the increased caspase-3 and synaptophysin. The data indicate that both extracts have neuroprotective effects against chronic constriction injury-induced neuropathic pain. The observed protective effects are partially mediated through attenuation of oxidative and nitrosative stress and suppression of both neuroinflammation and neuronal apoptosis, suggesting substantial activities of both extracts in amelioration of painful peripheral neuropathy.

## Introduction

Neuropathic pain is usually considered as a chronic pain condition and its affects about 3–18% of the population^1^. It is a serious neurological disease, resulting from the damage of a somatosensory nervous system, which consists of peripheral fibers (myelinated Aβ, Aδ and unmyelinated C fibers) and central neurons^2^. The etiology of neuropathic pain is diverse and includes physical injury, infection, metabolic or autoimmune disorders or any other condition that may result in a pathology of the nervous system. It is characterized by increased sensitivity to innocuous (allodynia) and noxious stimulation (hyperalgesia)^3^. Following the prolonged peripheral injury, profound central sensitization occurs^4^ and contributes to altered pain-related activity in neuropathic pain^5^.

There are many mechanisms underlying the pathogenesis of neuropathic pain, and ROS play an essential role in initiating and maintaining the pain. Peripheral nerve damage and cutaneous neurogenic inflammation elevated oxidative stress in the spinal cord with pain hypersensitivity as a consequence. Neural network is highly susceptible to ROS because of its increased lipid content. It was reported that CCI to the sciatic nerve lead to an increase of the endoneurial lipid peroxidation^6,7^. It is also known that ROS accumulation in the spinal cord activates several inflammatory mediators such as cytokines and prostaglandins which enhance the neuronal excitability^8,9^. In addition to the increase in peripheral nerves and spinal cord synaptic networks ectopic activity, the descending brain stem nociceptive circuits to the spinal cord are also activated, and play an essential role in pain-related behavior severity^6,10^. We previously showed that oxidative stress is elevated in the brain stem as well as in sciatic nerve, and is responsible for several conditions such as allodynia, hyperalgesia, and inflammation that follow the CCI of the sciatic nerve^6,10^, Moreover, we demonstrated that the centrally acting angiotensin receptor blocker, telmisartan had a favorable effect in improving the pain-related behavior in CCI compared to the peripherally acting one losartan^11^. Therefore, studying the supraspinal mechanisms involved in neuropathic pain in the current study is equally important.

The current treatments for neuropathic pain repose mainly on tricyclic antidepressants (tricyclics or TCAs), nonsteroidal anti-inflammatory medicines (NSAIDs), anticonvulsants, and opioids; however, many patients do not respond to these conventional medications^12^. They are also accompanied by side effects, such as sedation. So, understanding the mechanisms of action of effective alternative agents, endowed with analgesic properties is imperative to achieve ideal therapeutic efficacy.

Both *Thymus algeriensis* (TA) and *Thymus fontanesii* (TF), belong to the mint family Lamiaceae. They are widely distributed in Mediterranean basin. *T. algeriensis* is used in folk medicine to remedy respiratory problems (e.g., common cold), gastrointestinal disorders and prostate benign hypertrophy, and were also reported to abrogate miscarriage^13–15^. On the other hand, *T. fontanesii* is used as food preservative and to treat some gastrointestinal diseases^13^. We previously reported that both extracts possess analgesic, antipyretic, antioxidant, and anti-inflammatory effects. Moreover, both extracts inhibit the inflammation pathway. Extracts contain several secondary metabolites, among them flavones and flavonols such as luteolin, apigenin and quercetin, and phenolic acids, such as rosmarinic, phloretic and caffeic acids and their derivatives^13^. Previous studies showed that flavonoids and phenolic acids are beneficial against different models of neuropathic pain^6,10,14^. Interestingly, previous reports show that a number of *Thymus* species including TA exert acetylcholinesterase (AChE) inhibitory activities and hence they may be useful if used in different neurodegenerative disorders^14,15^.

The current work was undertaken to investigate the antioxidant activities of extracts of both species, *T. fontanesii and T. algeriensis* on human immortalized keratinocytes and their possible mechanism of action. This study aimed also at evaluating these medicinal herb extracts for their anti-inflammatory and antinociceptive effects in rats with CCI-induced neuropathic pain model, elucidating the molecular mechanisms involved both on the peripheral (sciatic nerve) and supraspinal (brain stem) levels.

## Materials and methods

### Plant material and extracts preparation

Leaves of *Thymus fontanesii* Boiss. et Reut. and *Thymus algeriensis* Boiss. et Reut. were collected from Algeria. Voucher Specimens (TA-L10, TF-L11) were deposited at the herbarium of the Pharmacognosy Department, Faculty of Pharmacy, Zagazig University, Zagazig (Egypt). Plant collection coordinates, identification and extraction were performed according to Sobeh et al.^13^.

### Cytotoxicity

All cell lines, with the exception of HaCaT cells (Innoprot, Berio, Spain), were from ATCC. Cells were cultured as previously described^16^. To evaluate the biocompatibility of the extracts, dose–response experiments were performed. 2.5 × 10^3^ cells per well (96-well plates) were seeded 24 h prior adding the extracts (10–100 µg/mL). Cells were then kept in an incubator set at 37 °C and 5% CO_2_ for 48 h, then, the MTT [3-(4,5-dimethylthiazol-2-yl)-2,5-diphenyltetrazolium bromide] assay has been used to analyze cell viability according to the method of Petruk et al.^16^. The cell viability is defined as follow: % Cell Viability = 100 *A/A_0_.

Where A and A_0_ corresponds to the viable cells in the presence of the extract and the negative control cells, respectively. The latter corresponds to untreated cells and cells incubated with buffer instead of extracts.

### Determination of the antioxidant capacity using a cell-based model

The DCFDA (2′,7′-dichlorofluorescein diacetate) assay was used to study the antioxidant activity of TA and TF extracts. HaCaT cells were seeded on plates at a density of 5 × 10^4^ cells/cm^2^. After incubation for 24 h, extracts were added to the plates at a concentration of 50 µg/mL and maintained for 120 min and then exposed to UVA radiations (100 J/cm^2^) to induce oxidative stress, as described before^17^. The HaCaT cells were then incubated with the probe (H_2_-DCFDA, Sigma-Aldrich, 20 µM) and the procedure described in^17^ was followed. Reactive oxygen species (ROS) levels were expressed as the ratio between DCF (2′,7′-dichlorofluorescein) fluorescence intensity determined for each sample, compared to the untreated cells.

### Western blot (WB) analyses

The HaCaT cells were first seeded at a density of 2 × 10^4^ cells/cm^2^ and incubated for 24 h. At the end of the incubation, each extract was added at a concentration of 50 µg/mL and then incubated for 15 or 30 min. The nuclear pellet was then obtained by centrifuging the cells and resuspending each pellet in RIPA buffer composed of 50 mM Tris–HCl pH8.0, 150 mM NaCl, 0.1% SDS, 1% NP-40, and proteases inhibitors. After centrifugation, 0.1 mg of the nuclear proteins (pellet) were used to conduct the WB analysis as previously described^16^. Specific antibodies (from Cell Signal Technology, Danvers, MA, USA) were used to detect the Nrf-2. The B-23 was used as internal standard (antibody from ThermoFisher, Rockford, IL, USA).

### In vivo* experiments*

#### Animals

Male Wistar rats used for this study weighted 275–300 g. These laboratory rats that were obtained at the Faculty of Veterinary Medicine, Zagazig (Egypt) were housed in plastic cages at constant environmental conditions (22 °C with 50 ± 10% humidity, 12 h:12 h light–dark cycle). They had also a regular chow diet ad libitum and free access to water.

#### Experimental design

The Ethics Committee of the Faculty of Pharmacy (ECAH ZU) at the Zagazig University (Egypt) has approved the investigative protocols that were carried out in compliance with the recommendations of the Weatherall study (approval number: ZU-IACUC/3/F/115/2018) and conducted following the guidelines of the US National Institutes of Health on animal care and use. The Wistar rats were allocated, randomly and equally (n = 6), to eight groups. Group 1 includes control animals that received the vehicle. Group2 is the sham operated group and received the vehicle. Group 3 includes CCI rats that received the vehicle. Groups 4–8 include CCI rats that received TA or TF at two concentrations (200 mg/kg and 400 mg/kg) or 30 mg/kg of pregabalin for rats that serves as positive control. Both plant extracts and the pregabalin were dissolved in dH_2_O using as suspending agent, 10% w/v of the acacia gum and given by oral gavage (5 mL/kg) daily for the whole period of the experiment (2 weeks) starting from the 1st day following the neuropathic pain induction.

#### Neuropathic pain induction

The CCI to sciatic nerve was used to induce the neuropathic pain as previously reported^11^. Briefly, following anesthesia with thiopental sodium (50 mg/kg, i.p.) the right sciatic nerve was exposed under aseptic conditions at the mid-thigh level beneath the gluteus and biceps femoris muscles. A common sciatic nerve segment (7-mm-long) proximal to the trifurcation was separated from the surrounding tissue. The CCI was then induced by placing four loose ligatures (4/0 silk suture) 1 mm apart around the nerve. The animals were returned to their cages following wound closure to recover.

#### Behavioral tests

Behavioral experiments were carried out by an experimenter who ignored the nature of the tests and was blinded to the different treatments.

##### Heat hyperalgesia

Heat hyperalgesia that refers also to the hot plate test was conducted to evaluate the thermal hyperalgesia according to the principal of Jain et al.^18^. Briefly, the rats were placed on a hot plate, maintained at a temperature of 52.5 °C ± 1.0 °C and the withdrawal latency to heat-induced nociceptive behavior was recorded with a cut-off time of 20 s.

##### Mechanical hyperalgesia (Pinprick test)

As detailed in our recent study^11^, a force was applied gently to the mid-plantar surface of the injured hind paw by bent gauge to avoid damaging the skin. Paw withdrawal duration (s) was recorded with a minimum value of 0.5 s (for the brief normal response) and a maximum value of 20 s.

##### Acetone drop test (paw cold allodynia)

The response of each rat to cold allodynia was tested by spraying the injured paw with acetone (100 μL). Based on graded to a four-point scale the response was graded as follows: 0, no response; 1, quick withdrawal or flick of the paw; 2, prolonged withdrawal or repeated flicking; and 3, repeated flicking with licking of the paw. The test was repeated for three times with 5 min gap in between. The final score is the total of the individual scores. The minimum score was 0, while the maximum possible score was 9.

##### Paint-brush test (mechanical dynamic allodynia)

A smooth paintbrush was used to produce a dynamic allodynic response by rubbing the plantar area of the injured paw five times with 5 s intervals and the number of withdrawals was recorded (between 0 and 5). The process was performed three times with a resting period of 5 min in between. A single cumulative score of the total number of withdrawals (in three tests) was calculated with a minimum value of 0 and maximum of 15^13^. Biochemical analysis in sciatic nerve and brainstem tissue were done as before^5^, and the methods were detailed in the supplementary file.

#### Histopathological studies

Sciatic nerves and brains were extracted from animals (n = 6 each) and fixed in 10% of neutral buffered formalin (NBF) and mixed into paraffin; sections at a thickness of 5 µm were then obtained and mounted on glass slides. The sections were first deparaffinized by treatment with xylene and then stained with hematoxylin and eosin (H&E stain). Histopathological examination was performed as before^19^. Methods of osmic acid stain of sciatic nerve and immunohistochemical studies were done as before in Sobeh et al.^5^ and the methods were detailed in the supplementary file.

### Statistical analysis

Biological replicates were analyzed in three independents replicates in *in-vitro* study. The used data are expressed as mean values ± SD. The significance point was fixed at 0.05 and the analysis were conducted using the Student's t-test. While in animal studies, data are expressed as the mean ± SEM. The data were subjected to one-way ANOVA or RM-ANOVA (repeated-measures analysis of variance). Differences among groups were determined by the Student's *t*-test and for the post hoc analysis, the Tukey multiple comparison was chosen. All analyses of the present study were performed using GraphPad Prism V. 6.01 (GraphPad Software, San Diego, CA). *p* < 0.05 is considered statistically significant.

## Results

### Cytotoxicity experiment

**Figure 1:**
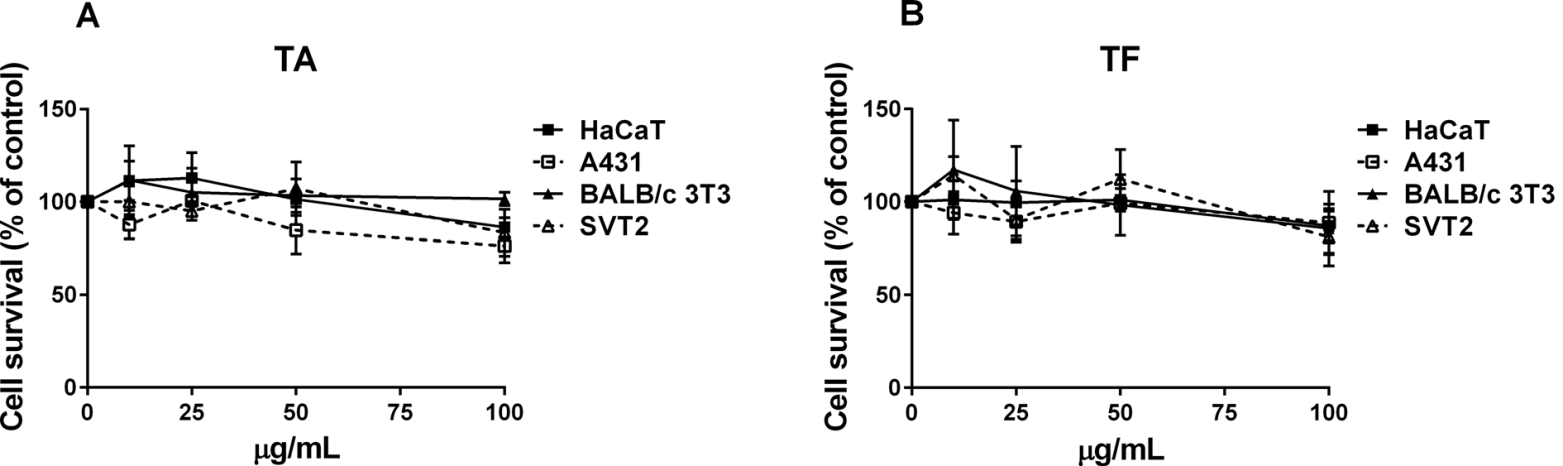
Immortalized and cancer cells viability after treatment for 48 h with increasing concentrations of *T. algeriensis* (TA) (**A**) and *T. fontanesii* (TF) (**B**) extracts (10**–**100 µg/mL). HaCaT (black squares), A431 (empty squares), BALB/c-3T3 (black triangles), and SVT2 (empty triangles) cells. Cell viability and cell survival were assessed according to method described in the section of materials and methods.

The cytotoxic activity of both extracts was evaluated on 4 cells lines, two cancer (A431 and SVT2 cells) and two immortalized (HaCaT and BALB/c-3T3 cells). After 48 h of cell incubation with increasing concentrations of each plant extract, the cytotoxic activity was measured, and the results are shown in Fig. 1. TA extract (Fig. 1A) was found to be fully biocompatible on both the immortalized cell lines tested and slightly toxic on cancer cells, but only at the highest concentration analyzed. TF extract showed the same activity on all the cell lines analyzed, with a very low toxicity at the highest concentration (100 µg/mL, Fig. 1B). Thus, 50 µg/mL of each extract were selected to test the protective effect against oxidative stress.

### In vitro* antioxidant activity*

**Figure 2:**
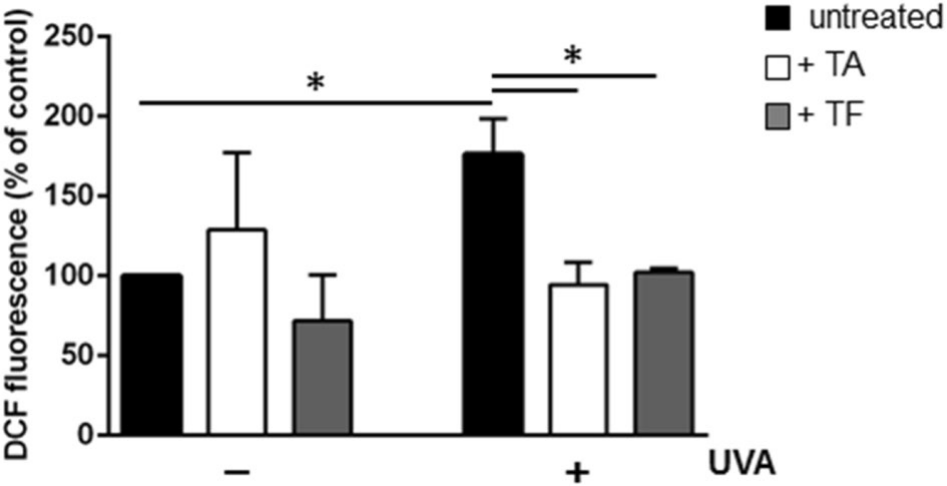
Antioxidant effect of TA and TF extracts (50 µg/mL) on UVA-stressed HaCaT cells. After 120 min of incubation with the *T. algeriensis* (TA) or *T. fontanesii* (TF) extracts, the HaCaT cells were stressed using the UVA (100 J/cm^2^). Untreated cells are reported as black bars. (−) indicates cells treated in the absence of stress, (+) indicates cells stressed by UVA. DCFDA assay was used to determine intracellular ROS levels. Statistical analysis was performed using one way-ANOVA, followed by Tukey post hoc test. Values are expressed as fold increase by comparing them with the control cells. *Indicates *p* < 0.05 compared to untreated cells.

To analyze the potential antioxidant effects of TA and TF extracts in vitro, HaCaT cells were challenged with UVA irradiation (100 J/cm^2^). As expected, UVA induced about a two-fold augmentation in intracellular ROS contents in comparison with the control (*p* < 0.05) (Fig. 2). However, our results showed that the incubation of the cells with TA (white bars of Fig. 2) or TF (grey bars of Fig. 2) extracts before UVA exposure lead to no significant modification in ROS levels.

### TA and TF extracts exert antioxidant effect by up-regulating Nrf2

**Figure 3:**
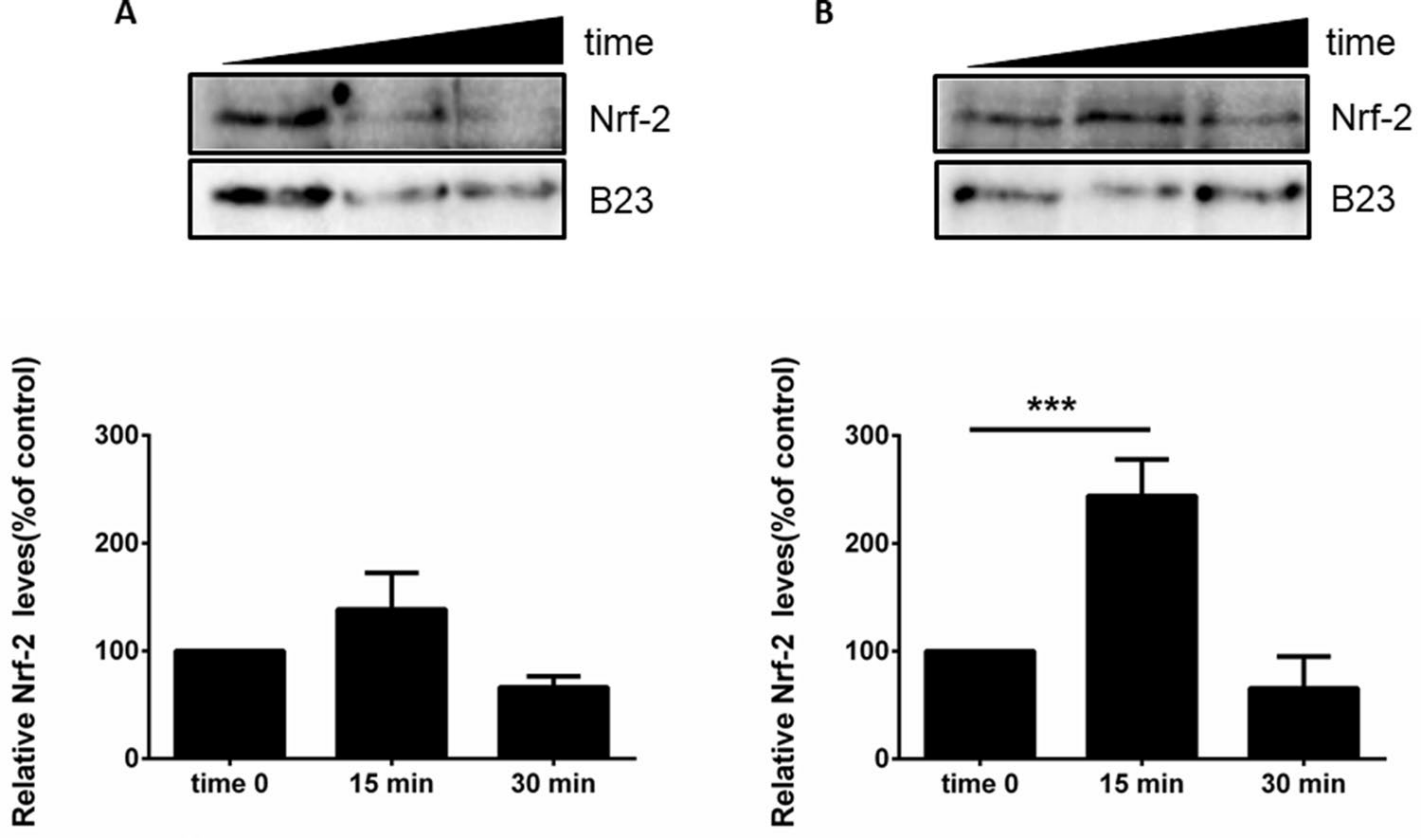
Effect of *T. algeriensis* (TA) and *T. fontanesii* (TF) extracts on the Nrf-2 levels of the HaCaT cells. After incubation of the HaCaT cells for 15 min (white bars) or 30 min (dark grey bars) with 50 µg/mL of either TA (**A**) or TB (**B**) extracts, nuclear proteins were extracted and used for Western blot analyses. The protein intensity levels were normalized to the internal standard (B-23) after being estimated by densitometric analysis. Statistical analysis was performed using one way-ANOVA, followed by Turkey’s post hoc test. ***Corresponds to *p* < 0.005 compared to control cells.

To help understanding the likely molecular mechanisms involved in the antioxidant effects of TA and TF extracts in HaCaT, we studied the implication of the Nrf-2 (nuclear factor erythroid 2) transcription factor, a key regulator of cellular antioxidant defense system that is highly expressed in epithelial cells including keratinocytes^20^. Kelch-like ECH-associated protein 1 (Keap-1) is normally associated to Nrf-2 to keep it in the cytoplasmic matrix and to direct it into the proteasome machinery for its degradation. Under stressful conditions, and/or in the presence of antioxidants, Keap-1, which is released from Nrf-2, translocates into the nucleus, and activates the transcription process of antioxidant genes that present in their promoter region, sequences named ARE (antioxidant responsive elements). As shown in Fig. 3, after incubation of the cells for 15 and 30 min with each extract, a significant increase in nuclear Nrf-2 levels was detected only after 15 min of incubation of the HaCaT cells with the TF extract.

### Effect of extracts on heat hyperalgesia and cold allodynia

**Figure 4:**
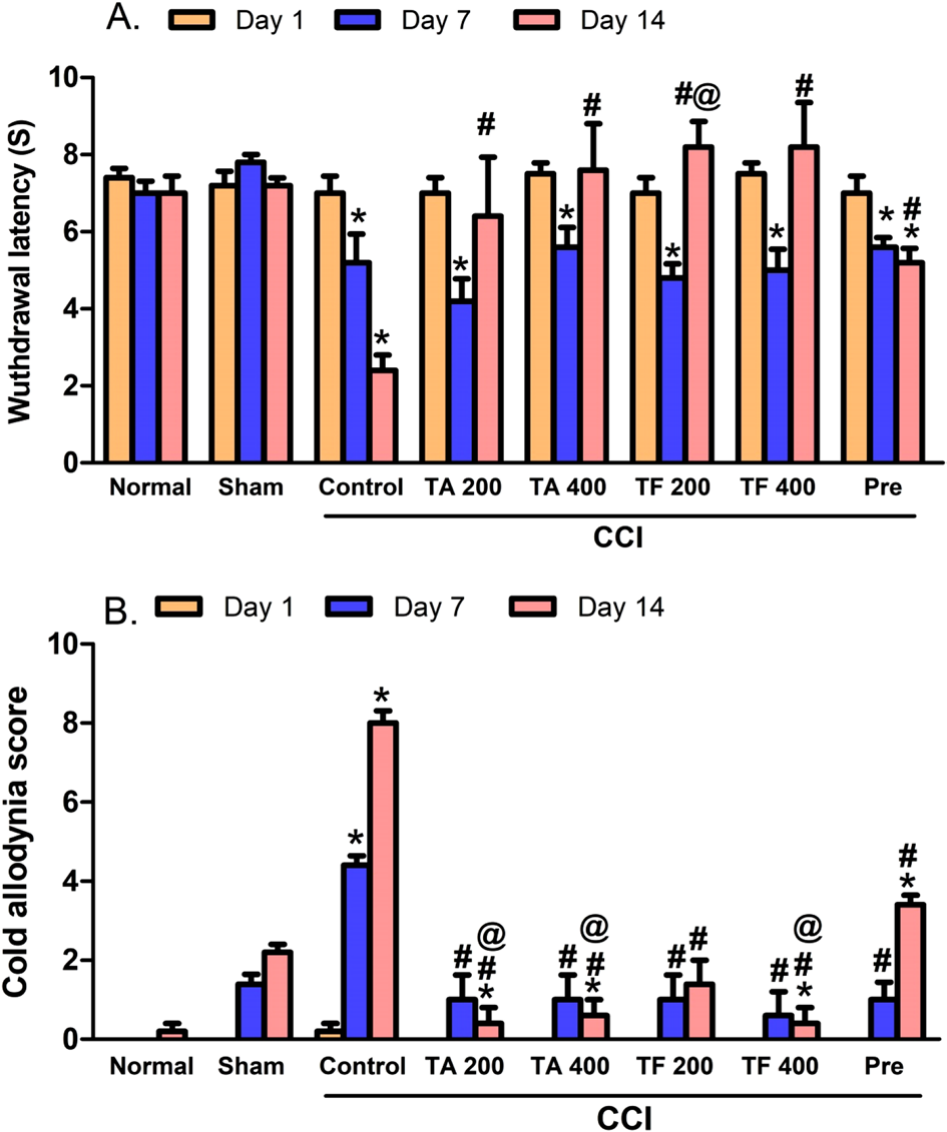
Effect of 200 and 400 mg/Kg of *T. algerensis* (TA) and *T. fontanesii* (TF) extracts against (**A**): heat hyperalgesia and (**B**): cold allodynia in neuropathic pain rats induced by spinal nerve ligation. Results are expressed as mean ± SE (n = 6). Statistical analysis was performed using RM-ANOVA, followed by Tukey post hoc test. The different symbols indicate the significance differences at p < 0.05 when compared with sham group (*), CCI group (#), and pregabalin group (Pre, ^@^) at the different time points as indicated in the materials and methods section.

Compared to the sham group, cold allodynia and heat hyperalgesia signs and were observed in rats exposed to CCI (Fig. 4A,B), these signs correspond to a time dependent decrease, cold allodynia scores and an increase in heat response latency time. Rats treated with 200 and 400 mg/kg of either TA or TF extracts revealed a restored heat response latency when measured at day 14 post CCI. Similarly, both extracts were able to restore normal cold allodynia responses starting day 7 post CCI, with. Noteworthy, for both cold and heat stimuli, the extracts showed superiority over the standard pregabalin drug, used for neuropathic pain, (Fig. 4).

### Effects of extracts on mechanical hyperalgesia and mechanical dynamic allodynia

**Figure 5:**
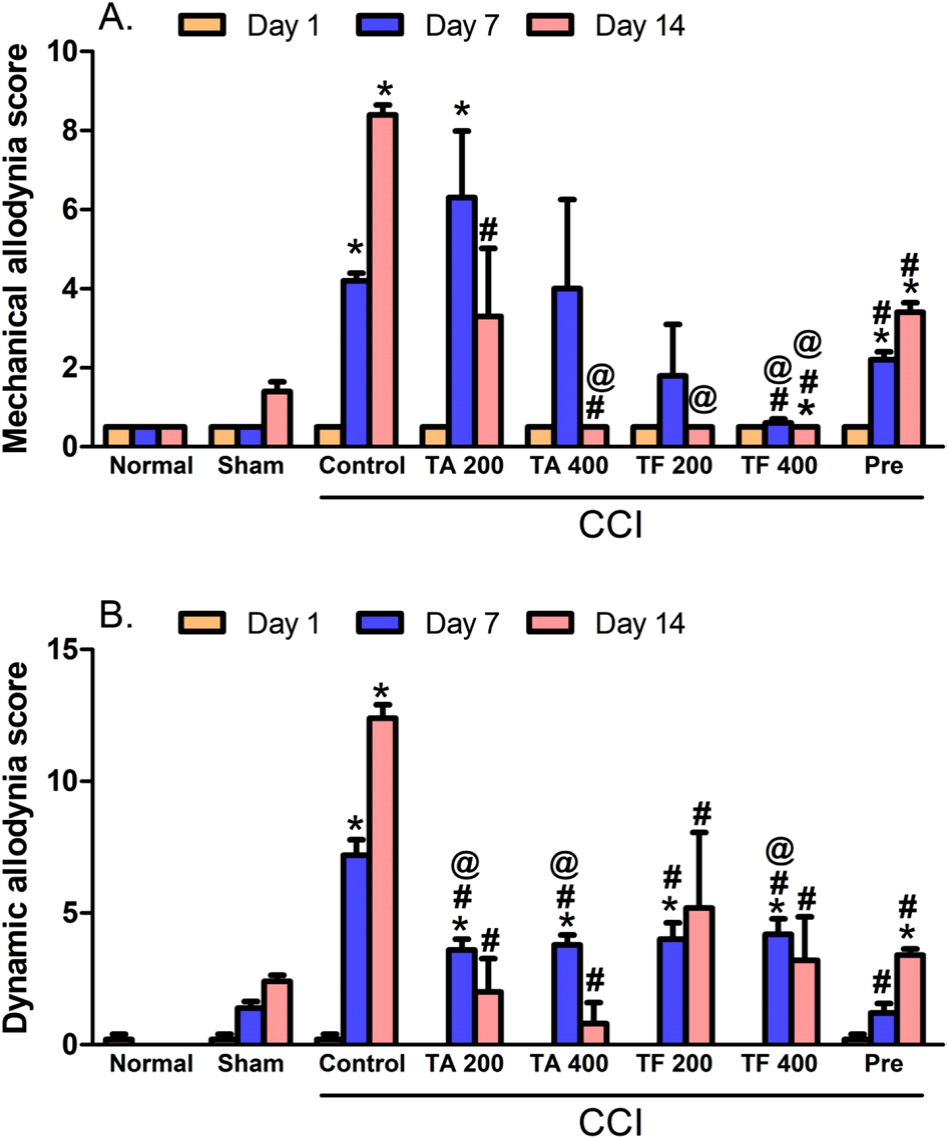
Effect of 200 and 400 mg/kg of *T. algerensis* **(**TA) and *T. fontanesii* (TF) extracts on (**A**): mechanical hyperalgesia score expressed as paw withdrawal and evaluated using the pin prick assay or (**B**): mechanical dynamic allodynia score (number of withdrawals) as evaluated in the neuropathic pain rats using the paint brush test. In response to the tip of the bent gauge needle, paw withdrawal duration in seconds (ranges from 0.5 s for the short normal response to a 20 s cut-off time), or the number of withdrawals (between 0 and 15) in a total of 15 experiments in response to a paint brush-induced stimulus were recorded and expressed as mean ± SE (n = 6). Statistical analysis was performed using RM-ANOVA, followed by Tukey post hoc test. The different symbols indicate the significance differences at p < 0.05 when compared with sham group (*), CCI group (#), and pregabalin group (Pre, ^@^) at the different time points as indicated in the materials and methods section.

Compared to the sham group, an increase by 8.4- and 6-folds after 7 and 14 days, respectively, was observed in the withdrawal time of injured hind paw (Fig. 5A). This results in a significant increase in the mechanical hyperalgesia in CCI rats This increase was attenuated or totally abolished when rats were given *T. algerensis* or *T. fontanesii* extract at all studied doses and the response was measured at day 14 post surgery (Fig. 5). As shown in Fig. 5B, CCI rats demonstrated higher dynamic allodynia (5 folds, *p* < 0.001)) scoring when evaluated by paint brush assay at the 7^th^ and 14^th^ day post-surgery with regards to the sham group. On the other hand, both extracts time dependently attenuated the dynamic allodynia response (Fig. 5B) as the later effect was highly attenuated when assessed at day 7 and totally abolished when measured at day 14 post surgery. Interestingly, *T. fontanesii* extract (400 mg/kg) has a greater effect against mechanical allodynia at day 7, compared to both *T. algerensis* and pregabalin.

### Histopathological changes

#### Effect of extracts on structural variations in sciatic nerve

**Figure 6:**
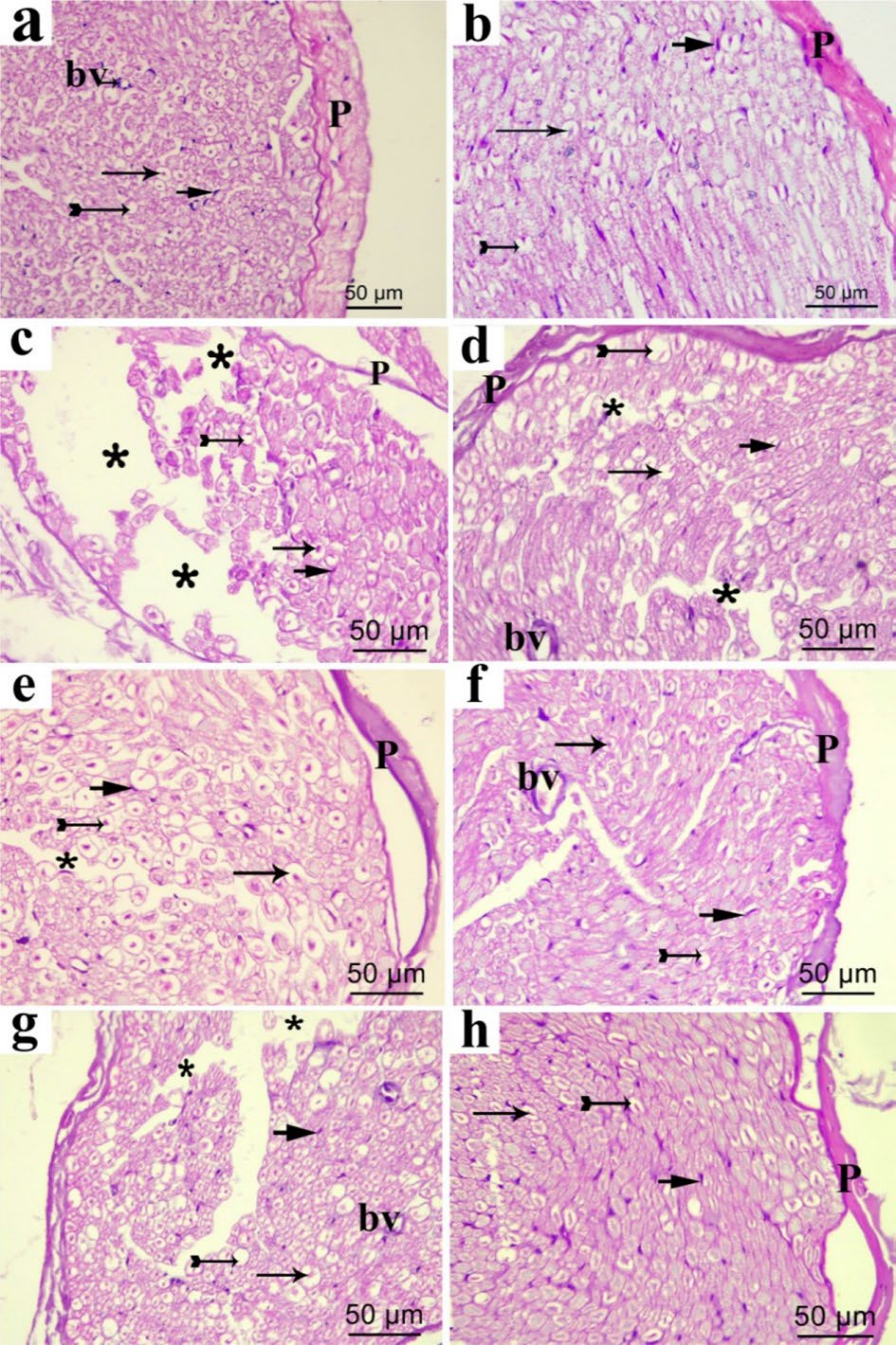
Representative photomicrographs of transverse sections of sciatic nerve in different experimental groups, (**a**) control group; (**b**) sham group; (**c**) CCI group; (**d**) pregabalin group; (**e**) TF (p.o., 200 mg/kg); (**f**) TF (p.o., 400 mg/kg). (**g**) TA (p.o., 200 mg/kg); (**h**) TA (p.o., 400 mg/kg). Arrow, bifid arrow, short arrow and astriex illustrate axoplasm, area of dissolved myelin, Schwann cells nuclei and wide separation between the nerve fibers, respectively. (P) perineurium; (bv) blood vessel. Scale bar, 50 μm × 400.

Histopathological analysis of the sciatic nerve tissues from all groups was performed using H & E and osmic staining (Fig. 6). Microscopic examination of the sciatic nerve transverse sections of H&E from control and sham group (Fig. 6a,b) revealed normal morphological appearance which showed the perineurium surrounding the closely packed nerve fibers of the nerve fascicle. Myelinated nerve fibers (abbreviated as MNF) are derived from axoplasm bounded by white patches of myelin and nuclei of the neurolemmocytes that appear between the nerve fibers. One will see an occasional endoneurial blood vessel (Fig. 6c). CCI group revealed disorganized nerve fascicles. Most nerve fibers are dissociated and are separated from their protective covering (perineurium). In pregabalin group and low doses of TF and TA groups (Fig. 6d,e,g), the nerve fascicle regained its normal form, However, the nerve fibers are still separated in some areas. By comparison, in the high-dose TF treated groups, apparently normal nerve fascicle consisted mainly of MNF and nuclei of the neurolemmocytes in endoneurial areas accompanied by minor differentiation in some few areas between the nerve fibers were seen (Fig. 6f). On the other hand, the high dose of TA restored the regular appearance of the nerve fascicle, which mostly consisted of neurolemmocytes nuclei and MNF in endoneurial areas without dissociation of the nerve fibers (Fig. 6h).

#### Osmic stain of sciatic nerve

**Figure 7:**
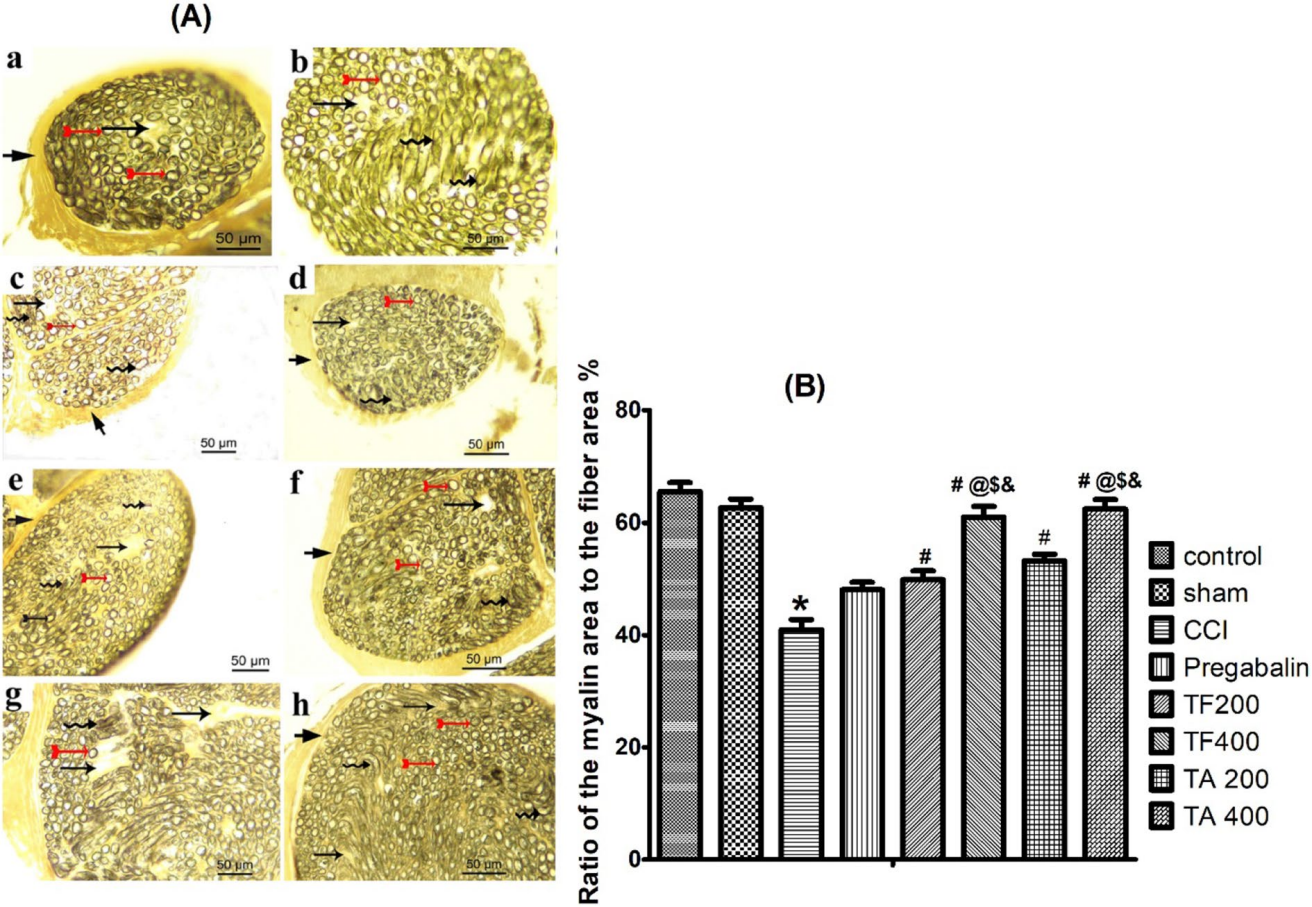
Representative Photomicrographs of osmic acid stained sciatic nerve transverse sections in (**A**): (a) normal group; (b) sham group; (c) CCI group; (d) pregabalin group; (e) TF (p.o., 200 mg/kg); (f) TF (p.o., 400 mg/kg). TA (p.o., 200 mg/kg); (f) TA (p.o., 400 mg/kg). Arrow, arrowhead, bifid arrow, short arrow and wavy arrow show the usual concentric lamellar structure of myelin sheath, unmyelinated nerve fibers, and irregular concentric lamellar structure of myelin sheath, respectively. Scale bar, 50 μm × 400. (**B**) Bar graph represents quantification of the ratio of myelin area to the fiber area %. Statistical analysis was performed using ANOVA one way, followed by post hoc testing by Tukey test. Values are set to mean ± SE (n = 6). ^*^Significant difference compared to the control group, *p* < 0.05; ^#^Significant difference compared to the CCI group, *p* < 0.05; ^@^Significant difference compared to the standard group, *p* < 0.05; ^$^Significant difference compared to the TF200 group, *p* < 0.05; ^&^Significant difference compared to the TA200 group, *p* < 0.05.

Osmic staining of sciatic nerves of different studied groups are shown in Fig. 7. In control and sham group (Fig. 7A-a,b), the nerve fascicle is surrounded by connective tissue perineurium and contain transverse section of myelinated nerve fibers of various sizes. Myelin sheaths appear as well-preserved darkly brown stained structures, often rounded or elliptical with endoneurium in between. In CCI group, the nerve fascicle showed irregular faint stained myelin sheath in many nerve fibers giving them distorted appearance with wide separation in between the fibers is observed (Fig. 7A-c). In pregabalin, low dose of TF and TA, sciatic nerves are still showing irregular myelin sheath in some nerve fibers giving them distorted appearance with slight separation (Fig. 7A-d,e,g). In contrast, high doses of TF and TA revealed nearly normal myelinated nerve fibers which are formed of darkly stained myelin sheath (Fig. 7A-f,h). We also showed that in normal nerve group and sham group, the myelin area to fiber area ratios were statistically similar (65.55 ± 1.59% and 62.64 ± 1.60%, respectively)). In contrast, in CCI group, the ratios of the myelin area to the fiber area were significantly decreased (40.95 ± 1.84%). Nonetheless, a significant increase in this ratio in pregabalin group (48.87 ± 1.36%), low dose TF group (49.86 ± 1.55%) and low dose TA group (53.22 ± 1.17%) was observed, they remain statistically different from the control. In contrary, in high dose TF group and high dose TA group, the myelin to the fiber areas ratio (60.98 ± 1.94 and 62 ± 1.67%, respectively) revealed significant increase compared to low dose TF group, low dose TA group and pregabalin group and were not significantly different from the control group (Fig. 7B).

#### Effect of extracts on structural changes of brain stem

**Figure 8:**
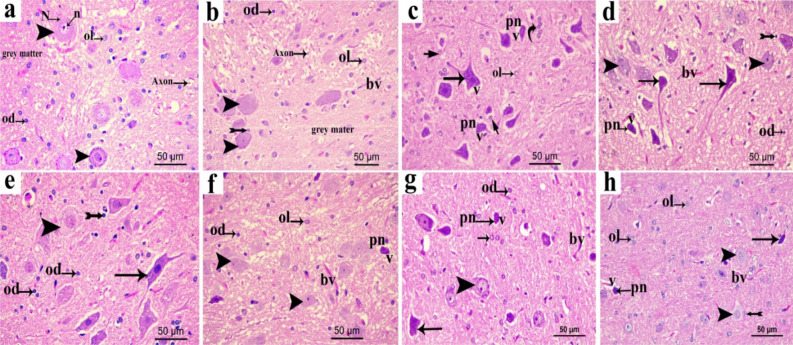
Representative photomicrographs in sections of brain stem from various groups (**a**) control group; (**b**) sham group; (**c**) CCI group; (**d**) pregabalin group; (**e**) TF (p.o., 200 mg/kg); (**f**) TF (p.o., 400 mg/kg). (**g**) TA (p.o., 200 mg/kg); (**h**) TA (p.o., 400 mg/kg). Arrowhead, arrow, short arrow bifid arrow illustrate normal neurons, affected neurons with dark stained cytoplasm and nuclei, perineural glial cell nuclei rarified lightly stained cytoplasm and nuclei, respectively.pn; pyknotic nuclei N; Nissl granule, n; nuclei bv, normal blood vessel; v; vacuolated cytoplasm, Ol: lightly stained glial cell, Od: deeply stained glial cell. Scale bar, 50 μm × 400.

Microscopic examination of the control groups of brain stem sections stained with the H&E stain (Fig. 8a) showed the gray matter with normal neuron having vesicular nuclei and basophilic cytoplasm containing Nissl bodies with prominent nuclei. Moreover, it was shown on the Fig. 8a that the acidophilic neuropil contains nuclei of normal neuroglia cells. The brainstem in sham group, is almost the same as control (Fig. 8b). The CCI group, on the other hand, showed degeneration of most neurons that had either shrunken vacuolated cytoplasm, with irregular and dark stained nuclei or pyknotic, small deeply stained nuclei with vacuolated cytoplasm. Glial cells with either lightly or deeply stained nuclei were seen. Perineural glial cells have been shown to be closely related to some degenerated neurons (Fig. 8c). Most neuron were degenerated and have pyknotic nuclei in pregabalin group, and few neurons were normal (Fig. 8d). Low-dose administration of TF (Fig. 8e) and TA (Fig. 8g) showed partial restoration of degenerative neuronal changes, predominantly TF where some neurons were regular, and some were affected with either large rarified lightly stained cytoplasm and nuclei or shrunken cytoplasm. Meanwhile, high-dose administration of TF (Fig. 8f) and TA (Fig. 8h) restored most of the degenerative neuronal changes that were more prevalent with high-dose TA (h) where most neurons were normal and few neurons exhibited pyknotic, low, deeply stained nuclei (Fig. 8).

### Immunohistochemical studies of brain stem

#### Effect of extracts on caspase 3

**Figure 9:**
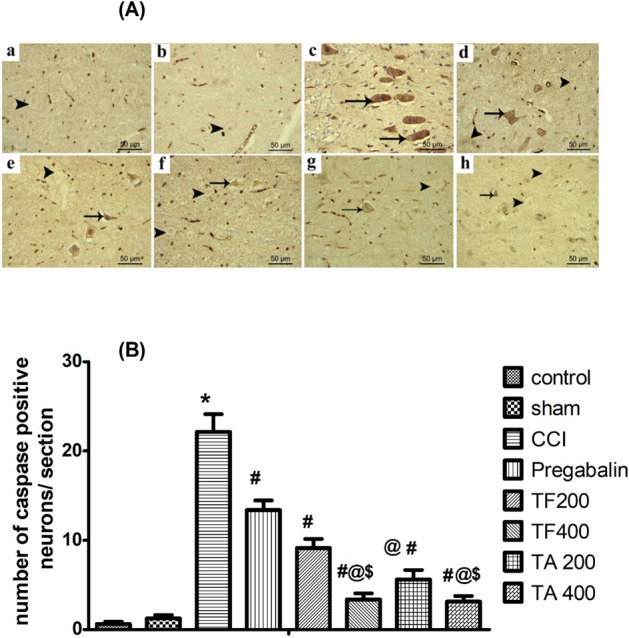
(**A**) Representative images showing the expression of the apoptotic neurons in different experimental groups through caspase- 3 immunostaining of brain stem sections (a) control group; (b) sham group; (c) CCI group; (d) pregabalin group; (e) TF (p.o., 200 mg/kg); (f) TF (p.o., 400 mg/kg). (g) TA (p.o., 200 mg/kg); (h) TA (p.o., 400 mg/kg). Arrow indicating dark brown staining of the cytoplasm of immunopositive neurons and arrowhead indicating the negative immunostaining neurons. Scale bar; 50 μm. (**B**) Bar graph showing differences in the number of caspase positive neurons in all study groups in the brainstem areas. The immunopositive neurons were counted over the entire 6 animals/group in 3 non-overlapping fields from × 200 magnification. Statistical analysis was carried out using one-way ANOVA, followed by a post-hoc test by Tukey test. Values are shown as mean ± SEM (n = 6).*Significant difference from the sham group, p < 0.05; ^#^Significant difference in comparison to the CCI group, *p* < 0.05; ^@^Significant difference compared to the pregabalin group, *p* < 0.05; ^$^Significant difference compared to the low dose TF group, *p* < 0.05.

Using an antibody anti-caspase 3, we immunohistochemically analyzed the brain stem tissues in order to determine the apoptotic neurons within the different studied samples. The caspase-positive neurons had been expressed as dark brown staining in the cytoplasm. They were negatively expressed in the control group (Fig. 9A-a) and sham group (Fig. 9A-b) which revealed non-significantly different results when compared with the control group. In contrast, caspase 3 immunopositive neurons in the CCI group (Fig. 9A-c) were apparently detected, showing a significant up-regulation in caspase 3 expression compared to the sham group. However, treatment with either pregabalin (Fig. 9A-d) or low dose of TF (Fig. 9A-e) tends to decrease the number of immunopositive neurons but still significant from the sham group. The number of immunopositive neurons revealed down-regulation in low dose of TA (Fig. 9A-g) which revealed significant difference from the sham group. Interestingly, the latter effect was better than the effect observed in the pregabalin group. On the other hand, treatment with high dose of TF (Fig. 9A-f) and TA (Fig. 9A-h) resulted in greater reduction in caspase 3 immunoreactivity and restored its level to the levels of the sham (Fig. 9).

#### Effect of extracts on synaptophysin (SYN) expression

**Figure 10:**
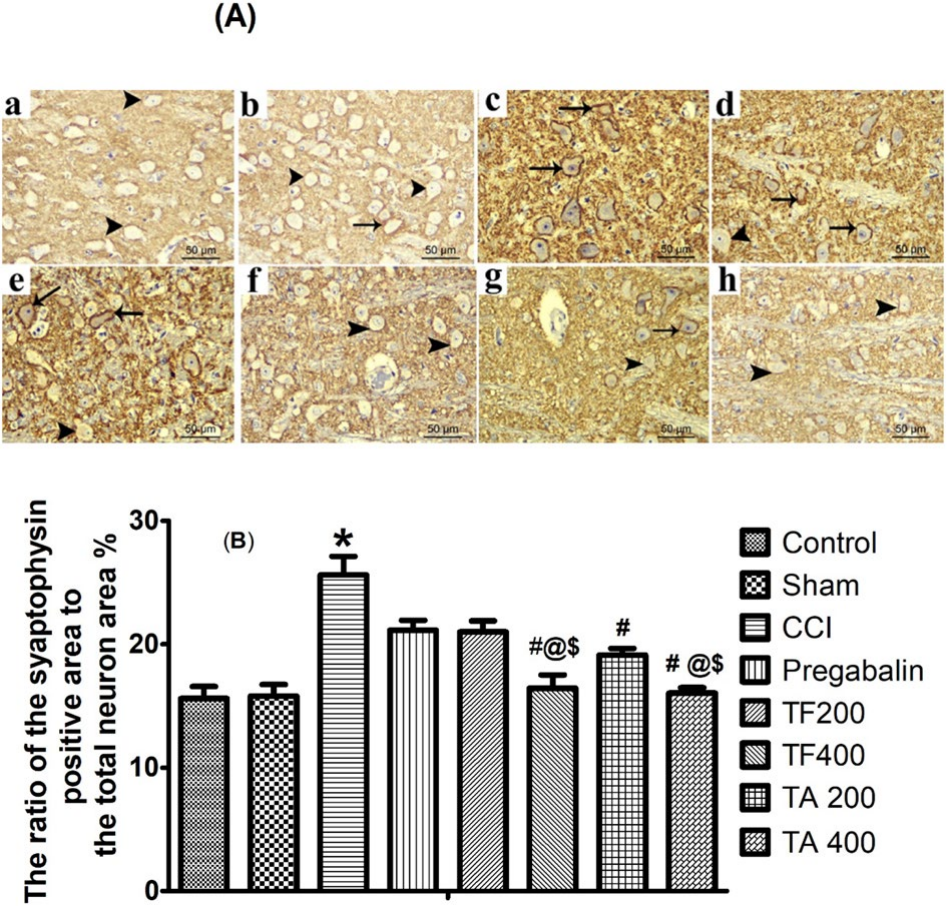
(**A**) Representative photomicrographs showing the expression of the synaptophysin in the neurons of sections in brain stem in different experimental groups. (a) control group; (b) sham group; (c) CCI group; (d) pregabalin group; (e) TF (p.o., 200 mg/kg); (f) TF (p.o., 400 mg/kg). (g) TA (p.o., 200 mg/kg); (h) TA (p.o., 400 mg/kg). Arrowhead indicating coarsely fine beaded reactivity at the neuron surface and arrow indicating dense bands of reactivity at the periphery of the neurons. Scale bar; 50 μm. (**B**) Bar chart showing the ratio of the synaptophysin positive reactivity on the surface of the neuron to the total area of the neuron in the brainstem sections of all experimental groups. Statistical analysis using one way ANOVA followed by Tukey post hoc test. Values are set to mean ± SE (n = 6). *Significant difference in comparison to the control group, *p* < 0.05**; **^**#**^Significant difference from the diseased group, *p* < 0.05; ^**@**^Significant difference compared to the pregabalin group, *p* < 0.05; ^**$**^Significant difference compared to the TF200 group, *p* < 0.05.

Synaptophysin reactivity was noticed, at the surface of the neuron in the brain stem of control and sham groups, under coarsely fine beaded reactivity form. As shown in the Fig. 10a,b, the dispersion of the reactive granules was also observed between the neurons. Chronic sciatic ligation resulted in an increase in SYN reactivity which was noticed in a form of dense bands at the periphery of the neurons and within the space between them (Fig. 10c). Treatment with either pregabalin or low dose of TF slightly decreased SYN immunoexpression. (Fig. 10d,e). However, Treatment with a higher dose of TF or TA (400 mg/kg) revealed a significantly lower SYN reactivity compared to lower doses (200 mg/kg).

The ratios of the synaptophysin positive reactivity on the surface of the neuron to the total area of the neuron were similar in both the control nerve group (15.60 ± 0.98%) (Fig. 10A-a) and in the sham group (15.79 ± 0.95%) (Fig. 10A-b). In CCI group (Fig. 10A-c)**,** the ratios of synaptophysin positive reactivity on the surface of the neurons to the total area of the neurons was significantly increased (25.61 ± 1.52%) compared to the control. However, pregabalin group (Fig. 10A-d) (21.14 ± 0.802%) and low dose TF group (Fig. 10A-e) (21.00 ± 0.89%) revealed significant decrease in the ratio compared to CCI group but still significantly different from the sham group. Interestingly, the use of high dose of either TF (Fig. 10A-f)**,** low dose TA (Fig. 10A-g)**,** or high dose TA (Fig. 10A-h) resulted in a greater reduction in the synaptophysin positive reactivity on the surface of the neuron to the total area of the neuron (16.43 ± 1.092%, 19.12 ± 0.55% and 16.04 ± 0.44%, respectively). Notably, the later effects were significant compared to CCI, TF and pregabalin groups, however, the differences were not statistically significant in comparison with the sham rats (Fig. 10B)**.**

### Effect of extracts on CCI-induced oxidative stress

**Figure 11:**
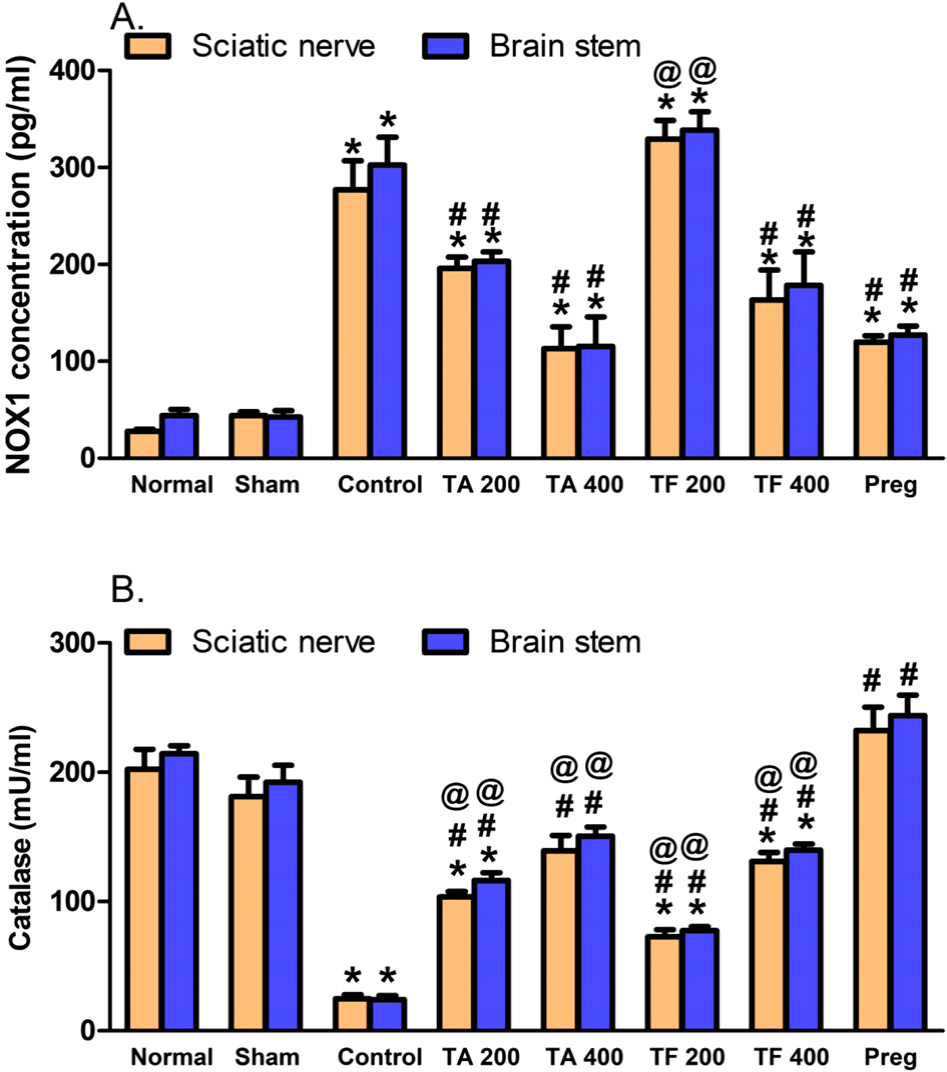
*T. algerensis* ( TA) or *T. fontanesii* (TF) extract (200 and 400 mg/kg, p.o) effects on CCI-induced increase in levels of NOX1 (**A**) and catalase activity (**B**) in the sciatic nerves and brain stems of CCI rats. Statistical analysis was performed using one way ANOVA, followed by Tukey post hoc test. Values are presented as mean ± SE, *n* = 5 rats per group. *p < 0.05, vs. Sham group; ^#^*p* < 0.05, vs. CCI group; ^@^*p* < 0.05, vs. pregabalin group (Pre). NOX1, NADPH oxidase.

The present study showed that the oxidative status was higher in the CCI rats' sciatic nerves and the brain stems (Fig. 11A). This increase was revealed by significantly (p < 0.0001) high NADPH oxidase (NOX1) contents and lower CAT activity in comparison with the sham group. However, groups with CCI treated with both doses of TA were able to improve their oxidative status through a decrease in the levels of NOX1 accompanied by a dose-dependent augmentation in the CAT activity in comparison with the sham rat values (Fig. 11A). However, TF extract (200 mg/kg) did not attenuate the increase in NOX1 level and mildly improved catalase activity. Doubling the dose of the extract led to a significant reduction in NOX1 level and elevation in catalase activity compared to CCI rats treated with the vehicle (Fig. 11B).

### Effect of extracts on CCI-induced increase in COX2, LOX and PGE2

CCI rats (14 days) showed substantially (*p* < 0.05) increased levels of sciatic nerve and brain stem COX2 (4.1 and 4.6 folds), LOX (4.2 and 3.95 folds) and PGE2 (2.8 and 3.1 folds) relative to sham tests, respectively. On the other hand, both TA and TF (200 and 400 mg/kg) attenuated inflammatory response after 14 days of treatment, rats showed a significant reduction in COX2 (58–65 and 22–62% respectively), LOX (40–52 and 35–59% respectively) and PGE2 (17–53 and 16–59% respectively ) compared to CCI control values. Noteworthy, TA reduces PGE2 levels to higher extinct compared to TF (Table 1).

**Table 1:** Effect of *T. algerensis* (TA) and *T. fontanesii* (TF) extracts (200 and 400 mg/kg) on different inflammatory markers.

Sample	COX2	LOX	PGE2	NF-kB	TNF-α	iNOS
	(ng/mL)		(pg/mL)			(U/mL)
**Normal**						
Sciatic	3.84 ± 0.40	0.84 ± 0.09	21.12 ± 1.28	49.04 ± 1.93	34.42 ± 2.99	3.60 ± 0.30
Brain stem	4.56 ± 0.49	0.99 ± 0.09	20.18 ± 1.31	57.48 ± 7.04	43.60 ± 6.11	3.75 ± 0.5
**Sham**						
Sciatic	6.22 ± 0.76	0.80 ± 0.09	24.68 ± 1.17	50.12 ± 3.54	38.76 ± 4.91	3.78 ± 0.47
Brain stem	6.92 ± 0.51	1.06 ± 0.09	24.54 ± 1.39	63.48 ± 3.35	52.12 ± 6.10	4.10 ± 0.43
**CCI**						
Sciatic	25.62 ± 1.15*	3.30 ± 0.40*	68.10 ± 6.58*	260.40 ± 13.31*	335.80 ± 26.66*	11.52 ± 0.78*
Brain stem	32.00 ± 1.64*	3.96 ± 0.29*	75.98 ± 7.11*	297.20 ± 22.14*	386.92 ± 27.25*	13.82 ± 0.44*
**TA (200 mg/kg)**						
Sciatic	10.75 ± 1.96*^#^	1.85 ± 0.19	56.82 ± 5.63*	78.20 ± 6.70^#^	54.65 ± 6.75^#@^	7.60 ± 1.94
Brain stem	21.00 ± 5.77*^@^	2.36 ± 0.25^#^	56.51 ± 8.27*	81.46 ± 7.97^#@^	58.43 ± 9.08^#@^	8.08 ± 1.33*
**TA (400 mg/kg)**						
Sciatic	8.92 ± 1.12^#^	1.98 ± 0.58	31.42 ± 3.85^#^	51.51 ± 6.34^#@^	31.80 ± 3.34^#@^	6.88 ± 1.57
Brain stem	13.41 ± 0.64^#^	2.14 ± 0.17^#^	31.18 ± 3.88^#^	55.38 ± 13.16^#@^	33.36 ± 4.25^#@^	6.60 ± 1.55*
**TF (200 mg/kg)**						
Sciatic	9.67 ± 0.64*^#^	1.83 ± 0.40	51.73 ± 6.73*	66.1 ± 3.37^#@^	60.20 ± 6.21^#@^	7.50 ± 2.27
Brain stem	24.84 ± 3.26*^@^	2.11 ± 0.65^#^	64.22 ± 12.43*	69.92 ± 2.62^#@^	65.55 ± 5.53^#@^	8.10 ± 2.36^#^
**TF (400 mg/kg)**						
Sciatic	10.68 ± 2.04*^#^	1.60 ± 0.38^#^	40.64 ± 11.28	41.12 ± 4.82^#@^	35.34 ± 3.27^#@^	9.68 ± 1.69
Brain stem	12.41 ± 0.62^#^	1.62 ± 0.49^#^	48.93 ± 4.58	44.60 ± 4.34^#@^	37.42 ± 4.66^#@^	10.33 ± 1.81^#^
**Pregabalin**						
Sciatic	10.64 ± 0.76*^#^	2.20 ± 0.27	32.30 ± 6.38^#^	114.08 ± 9.17*^#^	124.6 ± 4.27*^#^	7.28 ± 0.65*^#^
Brain stem	10.14 ± 0.86^#^	2.58 ± 0.17*^#^	47.00 ± 4.83*^#^	128.18 ± 10.19*^#^	139.38 ± 3.28*^#^	8.44 ± 0.63*^#^

Statistical analysis was performed using one way ANOVA, followed by post hoc testing by *Tukey* test.Values are presented as mean ± SE, n = 5 rats/group. *p < 0.05, vs. Sham group; ^#^*p* < 0.05, vs. CCIgroup. ^@^*p* < 0.05, vs. pregabalin group. COX2, cyclooxygenase 2; LOX, lipoxygenase PGE2, prostaglandin E2; NF-kB, nuclear factor kappa B; TNF- α, tumor necrosis factor alpha; iNOS, inducible nitric oxide synthase.

### Effect of extracts on NF-kB, TNF-α and iNOS

Sciatic nerve and brain stem of rats subjected to CCI for 14 days revealed significantly high levels of NF-kB (5 and 4.7 folds, respectively), TNF-α (8.7 and 7.4 folds, respectively) and iNOS (3 and 3.5, respectively) compared to sham rats. While, sciatic nerve and brainstem isolated from rats treated with *T. algerensis* or *T. fontanesii* extract (200 and 400 mg/kg, p.o, 14 days) showed abrogated CCI-induced increase in NF-kB and TNF-α and attenuated the increase in iNOS compared to CCI control group. Notably, while the effect of both extracts on NF-kB and TNF-α was superior to that of pregabalin, all treatments have similar effects on iNOS levels (Table 1).

## Discussion

Neuropathic pain, which can affect the peripheral and the central nervous systems, is always referred to a chronic pain condition that follows peripheral or central nerve injury caused either by trauma or systemic diseases such as diabetes, viral infection, multiple sclerosis and cancer. The available treatments for chronic neuropathic pain have limited efficacy in most patients^21^. Therefore, comprehensive studies are required to develop a better treatment for neuropathic pain. Here, we explored the possible protective effects of two species of thymus, *T. algerensis* (TA) and *T. fontansii* (TF) in a neuropathic pain rat model, sciatic nerve CCI model, and their mechanism of action. The main findings of our work are: (1) TA and TF administration substantially amended neuropathic pain behavior in the CCI model represented by hyperalgesia and allodynia induced by thermal and mechanical stimuli; (2) the structural derangements of both sciatic nerve and brain stem were improved following TA and TF treatments that was dose dependent and comparable to pregabalin in low doses and better than pregabalin at high doses; (3) both extracts improved synaptophysin expression in brain stem and suppress brain stem apoptotic marker caspase-3; (4) both extracts improved sciatic nerve integrity and maintain myelin sheath in CCI rats; (5) oxidative and nitrosative stress markers, such as iNOS, NOX1 and catalase were improved in both brain stem and sciatic nerve; (6) TA and TF extracts treatment protect HaCaT cell lines from UVA induced oxidative stress; (7) proinflammatory enzymes (COX-2 and LOX) and proinflammatory mediators (TNF-α ,NF-κB and PGE 2) were reduced after administration of both extracts in the brain stem and the sciatic nerve of CCI model rats.

The CCI model is a widely used neuropathic pain models. The peripheral nerve injury as a result of the sciatic nerve ligation leads to both structural and functional alteration in the injured nerve that can ultimately lead to both local inflammation and pain sensitization which peak two weeks following the nerve injury^22^. The ongoing peripheral damage is usually associated with central inflammation and sensitization; hence an effective therapeutic approach to treat neuropathic pain should target both the peripheral and central components of the disease.

Here, we demonstrated that rats developed both mechanical and thermal hyperalgesia in addition to the cold and dynamic allodynia especially when tested after days 7- and 14- post-surgery. In contrast to the study conducted by Chen et al. 2018^23^, the authors observed that hyperalgesia condition was obvious from day 3 to day 14 post CCI surgery of sciatic nerve with the peak on day 7. Our study showed that hyperalgesia condition appeared at day 7 and was more prominent at day14 post-surgery. The obtained results showed an attenuation of the CCI-induced neuropathic pain after the once-daily administration of both extracts. Their effects were dose dependent and superior to pregabalin, the reference standard used in the present study.

Intact peripheral nerves consist of resident macrophages, neurolemmocytes (or Schwann cells), fibroblasts, and the peripheral nervous system wrapping glia. Because, either central or peripheral components can orchestrate the neuropathic pain, we examined in our model, the effect of different treatments on the structural alterations induced by CCI in both the brain stem and the sciatic nerve. We were interested in studying brain stem area because several previous studies showed that chronic neuropathic pain is associated with ongoing functional alterations in the brain stem endogenous pain-modulation system^24,25^. Intact sciatic nerves consist of closely packed nerve fibers of the nerve fascicle that are surrounded by a perineurium. The myelinated nerve fibers are formed from axoplasm bounded by white of myelin and the nuclei of neurolemmocytes that appear between the nerve fibers. In the CCI model, the sciatic nerve is loosely ligated and chronically constricted. In the present study, H & E staining of the constricted sciatic nerves showed disorganization of nerve fascicles and wide separation of nerve fibers from each other and from overlying perineurium. Moreover, Osmic acid staining showed distortion of the myelin sheath in the CCI group. In previous studies, the afferent sensory neurons were reported to be first excited and the axons were then degenerated, resulting in a demyelination of these neurons^26^. This process causes the occurrence and establishment of peripheral neuropathy^27^. The present study showed that pregabalin and the low doses of TA and TF extracts established most of the normal situation of nerve fascicles, however, the nerve fibers remain separated in some areas and they did not reverse distortion of myelin sheath. On the other hand, high doses of TA and TF groups showed apparently normal myelin sheath and normal fascicles with slightly separated nerve fibers in few areas.

Both satellite glial cells and microglia, which represent the tissue-resident macrophages in the CNS, play an important role in restoring the physiological pain in sensory ganglia, particularly in dorsal spinal ganglia^27^. In the present study, we looked at brain stem that is responsible for a key role in the pain pathway. Our results showed degeneration and pyknosis of most neurons and the presence of perineural glial cells closely linked to other degenerated CCI neurons. Different previous papers indicated that glial cells are involved in neuron inflammation within the CNS^28^. Pregabalin and low doses of TA and TF extracts produce partial improvement, while high doses of extracts produce greater improvement.

Synaptophysin (SYN), the presynaptic protein marker, is localized in small synaptic vesicles and elevated in various neuropsychiatry disorders^29,30^. Our results indicated dendritic spine deterioration in brain stem following CCI due to alteration of expression levels of synaptic proteins, SYN. The expression levels of SYN in spinal cord were consistent with the duration of heat hyperalgesia caused by sciatic nerve CCI in rats revealing that CCI modulated SYN heat hyperalgesia^30^. Also, nerve transection significantly elevated the expression levels of SYN in the spinal cord horn^31^. The elevated SYN may be attributed to increased LOX and TNF-α observed in the present study. This note is supported by the finding that, 12/15-lipoxygenase overexpression elevates central SYN levels and triggers anxiety-related behavior and that TNF-α stimulates SYN expression in cultured endometrial stromal cells in a dose-dependent manner^29,31^. Both thymus extracts decreased synaptophysin expression in dose dependent manner with high doses exert more potent effect than pregabalin or low doses likely via attenuation of both LOX and TNF-α expression in brain stem.

Oxidative stress has been recognized as the contributor to chronic neuropathic pain. We showed previously that TA and TF have powerful antioxidant effects more than ascorbic acid *in vitro*^13^. In this work, we examined the TA and TF antioxidant effects, using the HaCaT cells exposed to UVA light. UVA light induced-oxidative stress was abolished by both extracts. This protective effect was due to Nrf-2 pathway in the case of TF extract. Nrf2 and its downstream pathway play an important role in maintaining the cellular redox status upregulated by means of different antioxidant enzymes, such as the NQO1 and HO-1. Further, we investigated the antioxidant potential of both extracts in CCI model. Our previous studies showed that catalase is decreased while, NADPH oxidase-1 (NOX-1) is increased in sciatic nerve and brain stem of CCI rats^6,10,11^. Catalase enzyme is one of the endogenous antioxidant enzymes present in peroxisomes and catalyzes the breakdown of H_2_O_2_ to H_2_O and O_2_. Excessive ROS formation in sciatic nerve and brain stem observed in our study may be responsible for the consumption of catalase activity 14 days post-surgery. Because of their antioxidant potential, both TA and TF extracts increased catalase activities in a dose dependent manner and give better effects than pregabalin. NOX-1 activation is the principal source of superoxide anion. Microglia, neurons, astrocytes, and macrophages in the dorsal root ganglion (DRG) and CNS expressed NOX1 during nerve injury^32^. ROS produced by NOX1 induction, in dorsal root ganglionic neurons, is responsible for pain amplification. Thermal and mechanical hyperalgesia was significantly attenuated in mice lacking Nox1^32,33^. The reduction of NOX-1 by both extracts may contribute to their protective effects against neuropathic pain. Our findings are consistent with those of a study that reported that TA extract has antioxidant potential and neuroprotective effect in a rat model of H2O2-induced brain injury^14^. According to previous studies, the sciatic nerve injury was reported to be responsible for the increase in gene expression of iNOS and NO in spinal cord microglial cells by the 5^th^ day post-surgery. We showed previously and in the current study that iNOS expression is also increased in sciatic nerve and brain stem 14 days post-surgery^6,10^. Activation of iNOS does not only increase the NMDA receptor GluN1 subunit phosphorylation but was reported to be also responsible for developing neuropathic pain following peripheral nerve injury^32–34^. Inducible NOS produces high concentration of NO which binds with superoxide anion produced by NOX-1 producing powerful peroxynitrite (PN) radical which causes different pain etiologies in the spinal and periphery cord^34^. The PN inhibits opioid signaling in the rostral ventromedial medulla (RVM) in the brain stem by nitroxidative modifications of endogenous opioid, enkephalin and formation of functionally inert neuropeptides thus causes chronic pain and central sensitization. Inhibition of peroxynitrite formation by inhibiting both NOX-1 and iNOS enzymes may contribute to the neuroprotective effects of TA and TF extracts.

Nitrosative products and ROS cause activation of NFκB and p38 via degradation /inhibition of IκB and MAPK phosphatases, that both causes inflammatory and neuropathic pain. Furthermore, NOX activity is essential for downstream NFκB- and p38 MAPK-dependent cytokine production. It is known that the inflammatory processes are mainly modulated by the NFκB factor, in glial and neuronal cells^35^. Our results showed an increase in the TNF-α (proinflammatory cytokine) and the NF-κB both in brain stem and sciatic nerve at the 14^th^ day post–surgery indicating a neuro-inflammatory response. Both TA and TF abrogated the increased NF-κB and TNF-α levels at all dose levels tested. Their effects were superior to pregabalin. It was shown that the pain behavior and the inflammation that follow the nerve injury were decreased in rats with transgenic inhibition of glial NFκB^35^. This result is in agreement with our finding findings.

High NFκB and TNF-α gene expression was linked to high LOX, COX-2 and PGE2 levels in sciatic nerve and brain stem 14 days post-surgery. Our findings are in accordance with others who reported that sciatic nerve injury is associated with higher NF-κB, TNF-α, LOX and PGE2 levels^36,37^. Notably, the changes in those inflammatory markers were abrogated by both TA and TF at all dose levels, an effect that was comparable to pregabalin. Our findings reveal that both extracts (TA and TF) are able to protect against peripheral nerve injury-induced neurogenic inflammation.

TNF-α binds to TNF-α receptor (TNFR) in Schwann cells causing the induction of apoptosis. The increased level of TNF-α causes intensification of neuronal death in the rat spinal cord. TNF-α was elevated around the injury area after spinal cord injury. We found that caspase-3, an apoptotic marker, was increased in brain stem of rats 14 days post-surgery. The increase in brain stem apoptosis observed in our rats is supported by others who reported that peripheral neuropathy is associated with neuronal loss and apoptosis in the rostral ventromedial medulla (RVM), a brainstem region involved in nociception^38^. The administration of TA and TF extracts decreased caspase-3 level in a dose-dependent manner. Notably, the effect of high doses was better than pregabalin. Collectively, our findings suggest that TA and TF exert anti-inflammatory and analgesic effects and protects against peripheral neuropathic pain injury. Moreover, both extracts can suppress neuronal cell death following nerve injury.

## Conclusions

*Thymus algeriensis* and *T. fontansii* extracts could effectively protect against painful peripheral neuropathy. Their underlying mechanisms may be through suppression of oxidative stress-induced neuroinflammation and apoptosis. Both extracts may be considered as promising therapeutic options for management of neuropathic pain and associated illnesses.

## Supplementary information

Supplementary Information.
