# Supplementary material for: Thymus algeriensis and Thymus fontanesii exert neuroprotective effect against chronic constriction injury-induced neuropathic pain in rats
Source: Sci Rep. 2020 Nov 25;10:20559. doi: 10.1038/s41598-020-77424-0 (PMC7688974; doi:10.1038/s41598-020-77424-0)
Supplement: Supplementary file 1 — Supplementary Information. [file 41598_2020_77424_MOESM1_ESM.docx]

***Thymus algeriensis* and *Thymus fontanesii* exert neuroprotective effect against chronic constriction injury-induced neuropathic pain in rats**

Samar Rezq^1^, Amira E. Alsemeh^2^, Luigi D’Elia^3^, Assem M. El-Shazly^4^, Daria Maria Monti^3^, Mansour Sobeh^5,^*, Mona F. Mahmoud^1,^*

^1^Department of Pharmacology and Toxicology, Faculty of Pharmacy, Zagazig University, Zagazig 44519, Egypt

^2^Department of Anatomy and Embryology, Faculty of Medicine, Zagazig University, Zagazig, Egypt

^3^Department of Chemical Sciences, University of Naples Federico II, Complesso Universitario Monte Sant’Angelo, via Cinthia 4, 80126 Naples, Italy

^4^Department of Pharmacognosy, Faculty of Pharmacy, Zagazig University, Zagazig 44519, Egypt

^5^AgroBioSciences Research Division, Mohammed VI Polytechnic University, Lot 660–Hay MoulayRachid, 43150 Ben-Guerir, Morocco

***** Correspondence: [mansour.sobeh@um6p.ma](mailto:mansour.sobeh@um6p.ma), [mfabdelaziz@zu.edu.eg](mailto:mfabdelaziz@zu.edu.eg)

Materials and methods:

Biochemical analysis in sciatic nerve and brainstem tissue

Following the last behavioral assessment, blood samples were collected via cardiac puncture under anesthesia (thiopental, 50 mg/kg, i.p.) followed by heart removal. The progression of NP involves both peripheral and central components, therefore, the anti-inflammatory potential of the extract was investigated in the sciatic nerve as well as in the brainstem, which is known to be greatly involved in maintenance of NP ^1,2^. Ipsilateral sciatic nerves, including the injured site and approximately 5 mm of surrounding tissues on both sides (~10 mm), were excised. Brainstems were then harvested. The collected tissues were either flash frozen in liquid nitrogen and maintained at -80 ^0^c or preserved in formalin for the histochemical analysis. Tissues homogenization was done in PBS followed by centrifugation (14,000 rpm for 20 min, 4 °C). Total protein of the supernatant was estimated using Bradford assay (Bio-Rad, 1000 Alfred Nobel Drive, Hercules, California 94547, USA). NADPH oxidase 1 (NOX1), cyclooxygenase-2 (COX-2), 5-lipoxygenase 1 (5-LOX), catalase, TNF-α, and NF-κB were detected using rat ELISA kits obtained from Cusabio (TX, USA), while Inducible nitric oxide synthase (iNOS) and Prostaglandin E2 (PGE2) were determined by ELISA kits from MyBiosource (San Diego, CA, USA) and Cayman (Michigan, USA), respectively, according to the manufacturers’ instructions^3,4^.

Immunohistochemical staining of brain stem synaptophysin and caspase-3

Paraffin sections of perfused brain stem from all studied groups were used for detecting, the synaptic protein, synaptophysin (SYN), and the apoptotic marker, caspase -3, immunoreactivity. The sections were de-waxed and incubated for 1hr at room temperature in 0.3% hydrogen peroxide in phosphate-buffered saline, pH 7.6 (PBS). The slides were washed 3 times (10 min. each) in the same buffer to quench endogenous peroxidase activity. They were incubated for 16 hr at 4 °C in PBS containing 2% normal goat serum (NGS) and 0.5% triton X-100, and then washed again in PBS at room temperature. This was followed by overnight incubation at 4°C with the primary monoclonal antibody of anti-SYN (1:150, CST, MA, USA) or anti-caspase-3 (1:500, CST, MA, USA), then washed 3 time in PBS-2% NGS. The primary antibodies were bounded by a rat adsorbed biotinylated anti-mouse secondary antibody (1:200, Vector Labs, Burlingame, CA, USA), in PBS for 1hr at room temperature. Slides were incubated in avidin-biotin complex linked to peroxidase (ABC Kit, Vector Labs, Burlingame, CA, USA). Peroxidase was seen with 0.03% diaminobenzidine hydrochloride and 0.005% hydrogen peroxide in 0.1 M Tris buffer. All sections were counter stained with hematoxylin dehydrated, cleared, and mounted in Canada balsam. The Image J analysis software (Fiji Image J; 1.51 n, NIH, USA) was used to estimate the ratios of the synaptophysin positive reactivity on the surface of the neuron to the total area of the neuron and to count the immunopositive caspase-3 nuclei^3,4^.

2.5.8. Sciatic nerves staining with osmic acid

For the morphometrical assessment of the sciatic nerve, osmic acid was used to stain the myelin sheath of the nerve, as previously described^3,4^

. 3-µm sections were fixed with 4% paraformaldehyde, incubated with 2% osmic acid solution for 3 days, transferred to 75% alcohol (2 h) then embedded in paraffin. Myelin sheaths were stained with a dark brown color upon examination by light microscopy (LEICA ICC50 W, Leica Microsystems (Schweiz) AG, 9435 Heerbrugg, Switzerland). The ratio of the myelinated area to the total nerve fibre area (including the myelin sheath) was detected and represents the integrity of the sciatic nerve. Additionally, the percentage of degenerated nerve fibers (fibers that lack the normal concentric lamellar structure of the myelin sheath or has myelin invagination into the axon and axonal swelling) to the total count of nerves was also estimated by a histologist who did not know the treatments^1^.


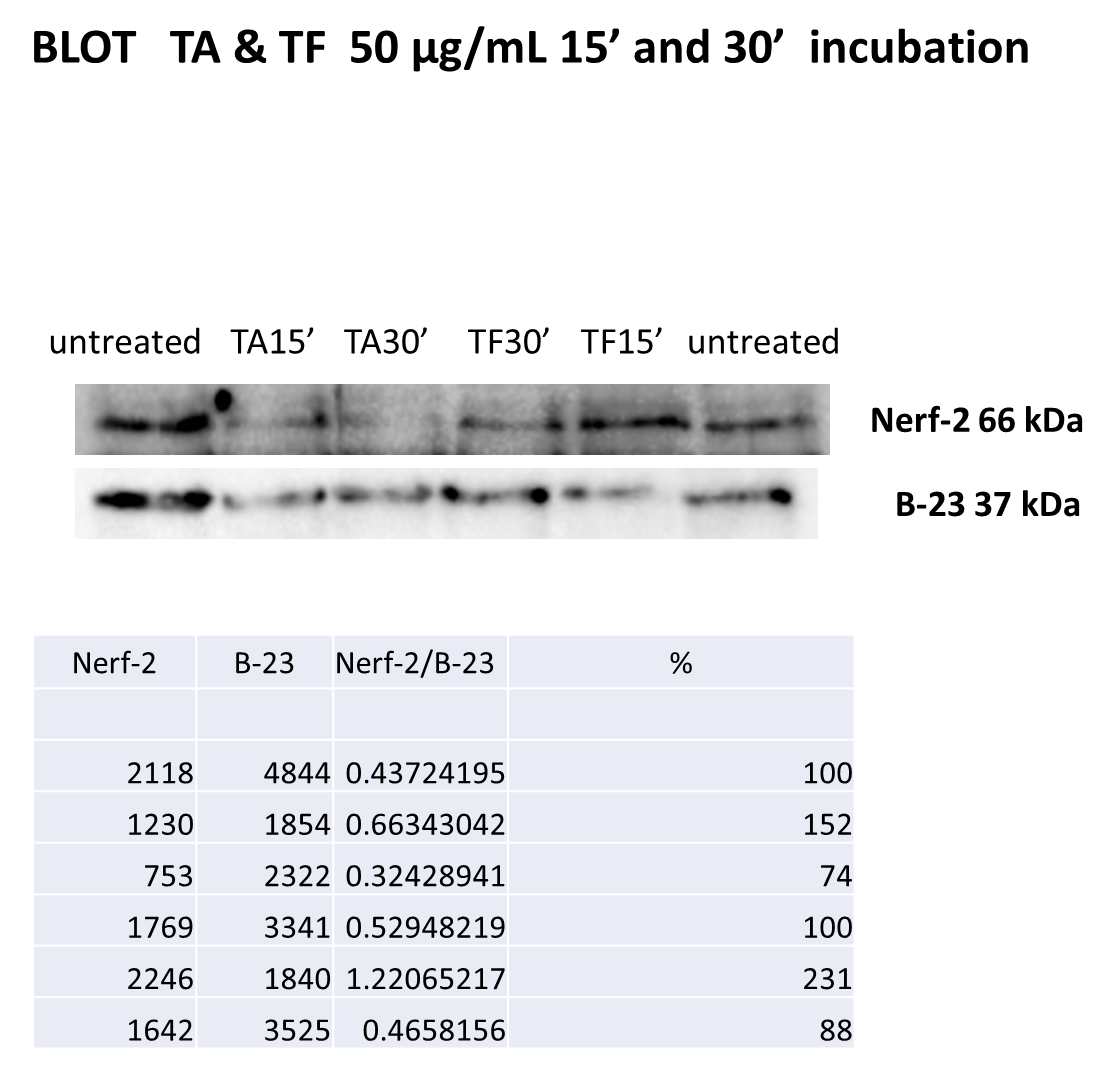


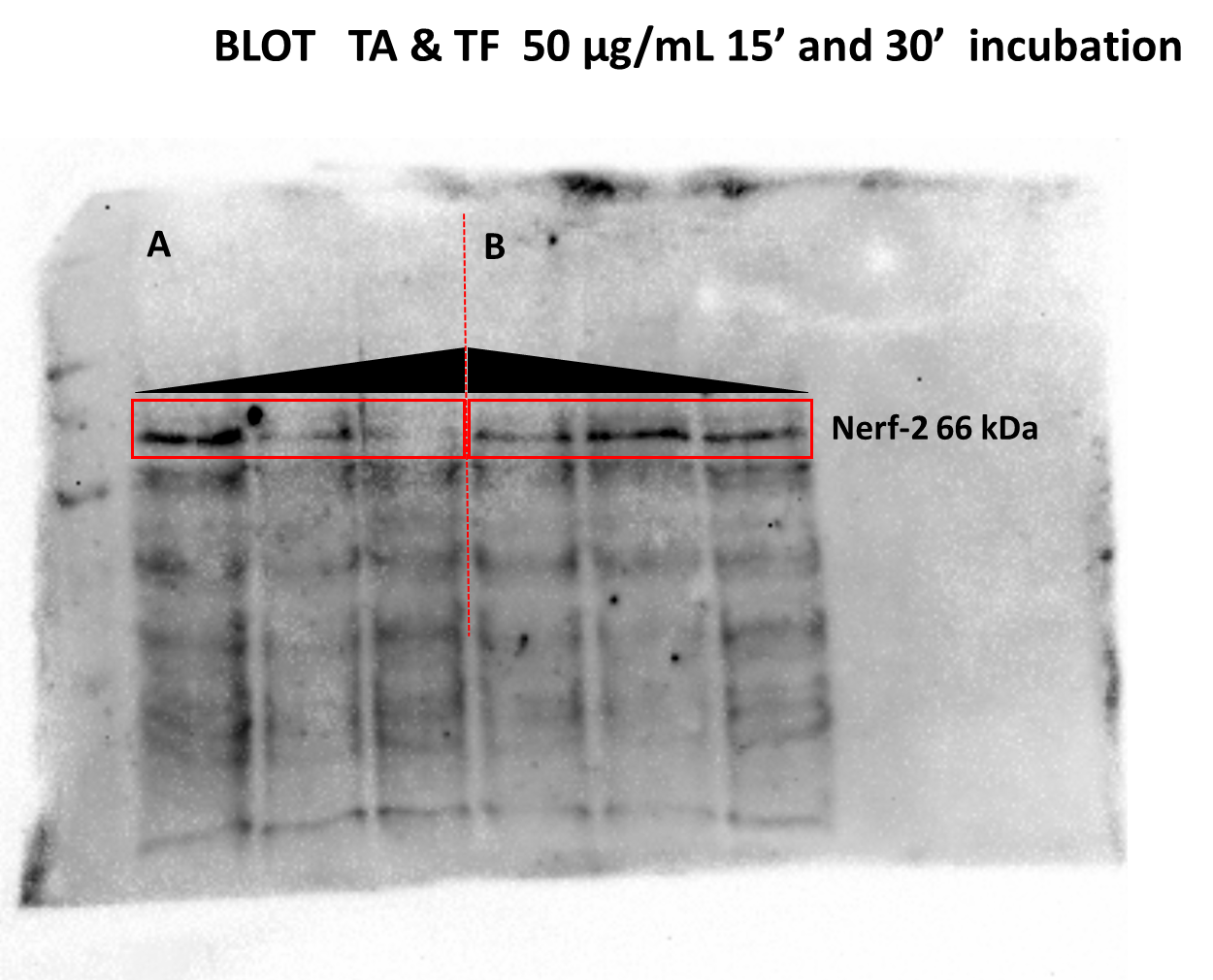


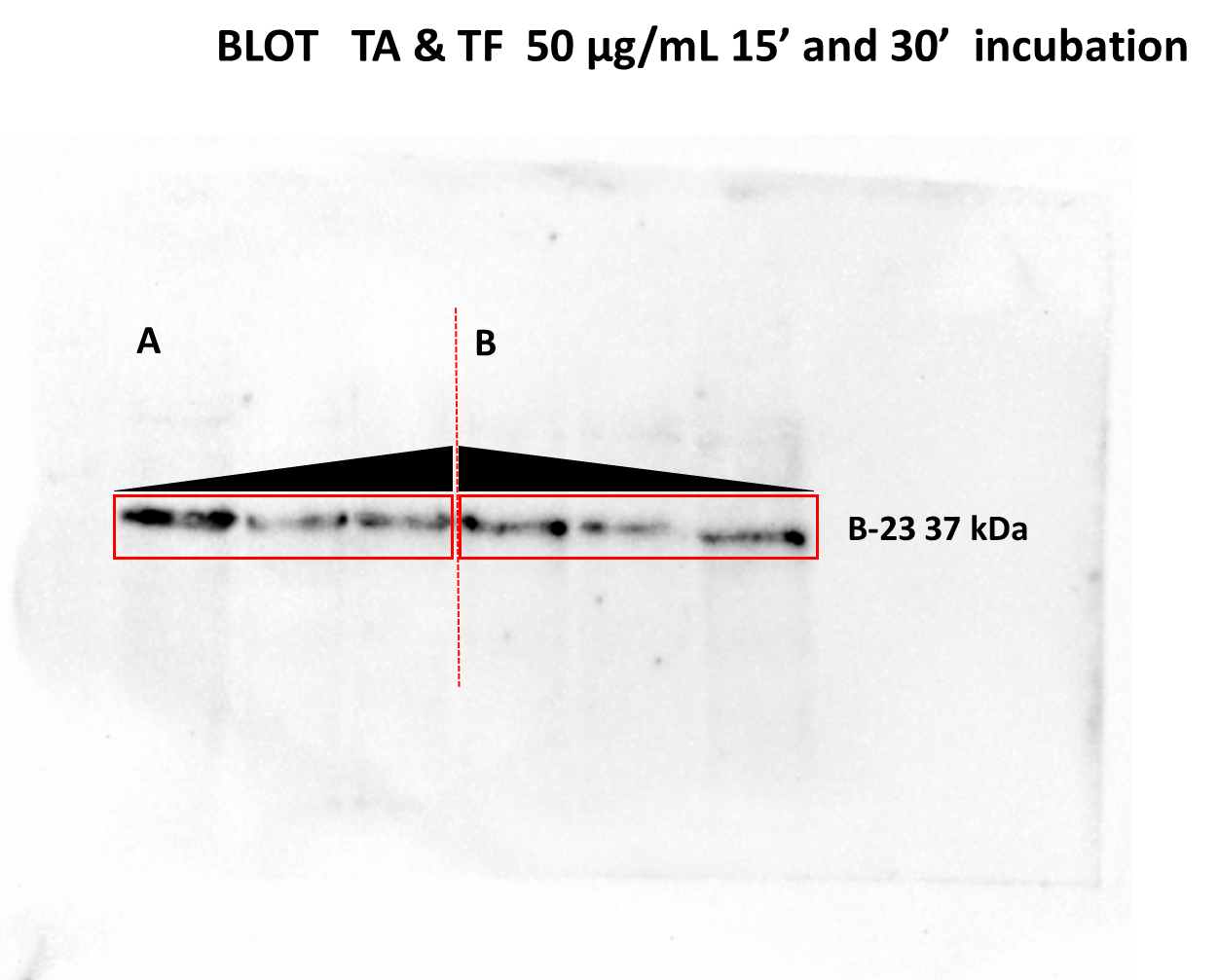


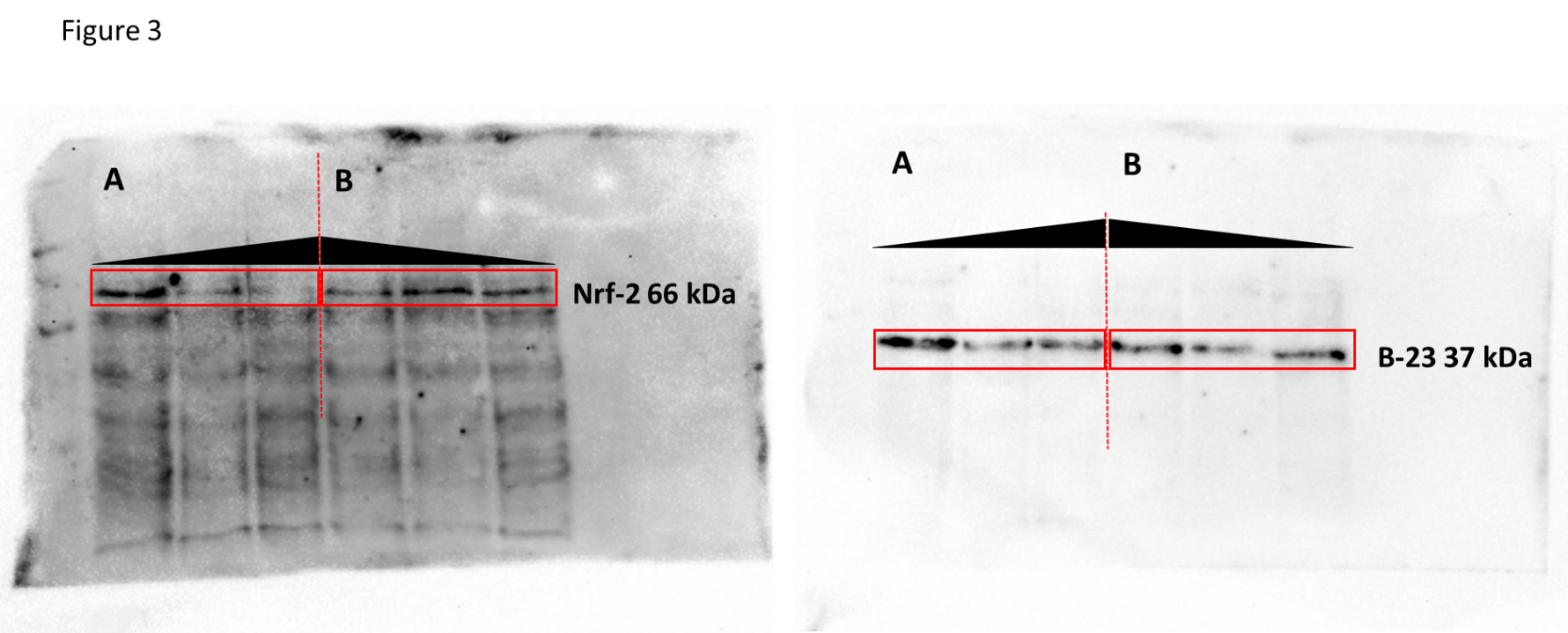


**References**

1. Colloca, L. *et al.* Neuropathic pain. *Nature reviews Disease primers* **3**, 1–19 (2017).

2. Mills, E. P. *et al.* Brainstem pain-control circuitry connectivity in chronic neuropathic pain. *Journal of Neuroscience* **38**, 465–473 (2018).

3. Sobeh, M. *et al.* Salix tetrasperma Roxb. extract alleviates neuropathic pain in rats via modulation of the NF-κB/TNF-α/NOX/iNOS pathway. *Antioxidants* **8**, 482 (2019).

4. Sobeh, M. *et al.* Haematoxylon campechianum Extract Ameliorates Neuropathic Pain via Inhibition of NF-κB/TNF-α/NOX/iNOS Signalling Pathway in a Rat Model of Chronic Constriction Injury. *Biomolecules* **10**, 386 (2020).
